# Genome Mining as New Challenge in Natural Products Discovery

**DOI:** 10.3390/md18040199

**Published:** 2020-04-09

**Authors:** Luisa Albarano, Roberta Esposito, Nadia Ruocco, Maria Costantini

**Affiliations:** 1 Department of Marine Biotechnology, Stazione Zoologica Anton Dohrn, Villa Comunale, 80121 Napoli, Italy; luisa.albarano@szn.it (L.A.); roberta.esposito@szn.it (R.E.); nadia.ruocco@gmail.com (N.R.); 2 Department of Biology, University of Naples Federico II, Complesso Universitario di Monte Sant’Angelo, Via Cinthia 21, 80126 Napoli, Italy

**Keywords:** bacteria, fungi, genome mining, natural products, synthetic biology

## Abstract

Drug discovery is based on bioactivity screening of natural sources, traditionally represented by bacteria fungi and plants. Bioactive natural products and their secondary metabolites have represented the main source for new therapeutic agents, used as drug leads for new antibiotics and anticancer agents. After the discovery of the first biosynthetic genes in the last decades, the researchers had in their hands the tool to understand the biosynthetic logic and genetic basis leading to the production of these compounds. Furthermore, in the genomic era, in which the number of available genomes is increasing, genome mining joined to synthetic biology are offering a significant help in drug discovery. In the present review we discuss the importance of genome mining and synthetic biology approaches to identify new natural products, also underlining considering the possible advantages and disadvantages of this technique. Moreover, we debate the associated techniques that can be applied following to genome mining for validation of data. Finally, we review on the literature describing all novel natural drugs isolated from bacteria, fungi, and other living organisms, not only from the marine environment, by a genome-mining approach, focusing on the literature available in the last ten years.

## 1. Introduction on Bioactive Natural Products Isolation

Nature is an important source of bioactive products and their derivatives (secondary metabolites), which form part of many important drugs formation widely used in the clinic field [1]. In fact, as reported in Newman and Cragg [2], over the last 30 years the great majority of anticancer, anti-infective, and anti-bacterial drugs are represented by natural products and their derivatives, produced by all organisms (from bacteria to plants, invertebrate, and other animals) with different chemical structure and leading to several biological activities [3,4]. Furthermore, these secondary metabolites have influenced the development of several drugs, including antibacterial, anticancer, and anti-cholesterol agents [5]. Several of these bioactive products are derived from microorganisms, such as fungi and bacteria [6], which have represented an important source of antibiotics and many other medicines [7,8]. In particular many bacteria deriving from the marine environment, particularly those found in association with marine invertebrates (such as sponges), are able to produce secondary metabolites with potential anticancer and antifungal roles because of their cytotoxic properties [9,10]. Considering the great problem of the antimicrobial resistance increase and its high impact on human health, there is an important need of searching for new natural products that could therefore remedy this issue [11,12]. For these reasons, in the past decade, genomic science has been used to identify the possible drug targets and to find novel genes cluster for the biosynthesis of natural products [13]. The development of the genome sequencing technologies to find novel metabolites has surely drown the attention of pharmaceutical industries, which had by now lost interest in natural products due to the advent of combinatorial chemistry [14]. The advent of based-genome sequencing techniques, especially with establishment of genome mining, has allowed to obtain new natural drugs in a faster and cheaper way.

### Genome Mining

**Figure 1:**
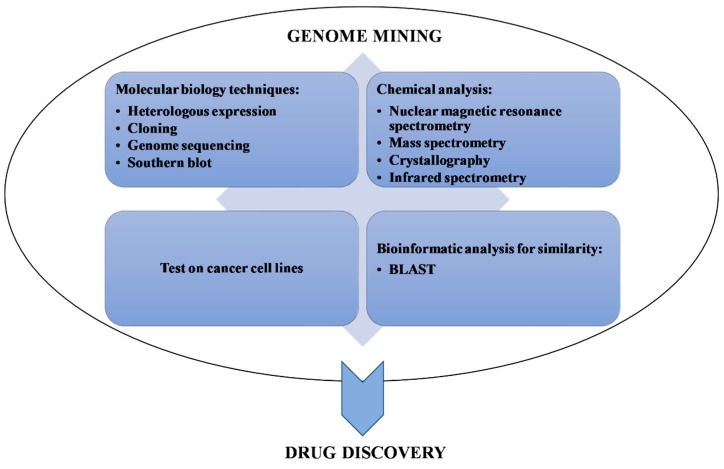
Associated techniques (categorized as molecular biology techniques, chemical analysis, cellular biology techniques, and bioinformatic analysis) to genome mining for validation of data, leading together to drug discovery.

The term “genome mining” are associated to every bioinformatics investigation used to detect not only the biosynthetic pathway of bioactive natural products, but also their possible functional and chemical interactions [15]. Specifically, the genome mining involves the identification of previously uncharacterized natural product biosynthetic gene clusters within the genomes of sequenced organisms, sequence analysis of the enzymes encoded by these gene clusters, together with the experimental identification of the products of the gene clusters (Figure 1; [16]).

Genome mining is entirely dependent on computing technology and bioinformatics tools. About this point, a huge amount of data, consisting of DNA sequences and their annotations, are now deposited in publicly accessible databases. The storage and handling of these resources relies on the continued development of computers and the networks. Once all the genes within a new genome are identified, they can be compared with those of known functions in the public databases. Both raw and annotated genomic data, as well as bioinformatics tools, for sequence comparisons are freely available through the different websites. It also important to keep in mind that it is now a mandatory publication prerequisite of most scientific journals that sequence data from research involving novel DNA sequences is deposited in a publicly accessible database.

In the case for which the sequences of many proteins, encoding for enzymes, involved in natural product biosynthesis are deposited in these databases, it is relatively easy to identify pathways in which they are involved by sequence comparisons. The availability of these synthesis enzymes and the pathways in which they operate, together with the sequence comparisons with genes from which they arise, can certainly be used to identify homologs, and potentially the pathways, in the new organism under analysis. However, it is important to consider that many enzymes are similar in sequences but follow chemical processes that are slightly different, leading to a different pathway or very different final end product.

Furthermore, genome mining has a strong support by synthetic biology, consisting in the design and the construction of new biological, as for examples enzymes, genetic circuits and/or the redesign of existing biological systems. These combined approaches are mainly used to detect novel natural products in bacteria and fungi probably because of operon organization of their synthesis genes [13], allowing the control of transcriptional levels and also the association of their potential metabolic function [17]. Moreover, the central role of genome mining consists in finding new biosynthetic gene clusters (BGCs). In fact, the BGCs encode for two class of enzymes, polyketide synthases (PKS) and non-ribosomal peptide synthases (NRPS), which are the two most important biosynthetic routes responsible for the formation of natural products [18]. This approach also provides the possibility to compare target gene clusters to known gene clusters useful for the prediction of their function and structure using different associated web database [5]. In fact, although the genome mining allowed to find and identify the gene clusters responsible for the production of natural product synthesis, in the last decade web tools and databases have been integrated to improve the performance of this approach [15]. This scientific progress has enabled the development of three important web tools: (i) “antibiotics and Secondary Metabolite Analysis SHell” (antiSMASH), its first version was issued in 2011 and it is a web server able to associate the gene clusters identification with a series of specific algorithms for compounds analysis [19]. Therefore, this approach performs the prediction of sequences and offers a more detailed analysis of identified gene clusters and consequently gives the predicted image of amino acid stereochemistry structure [5]. (ii) “PRediction Informatics for Secondary Metabolomes” (PRISM), open-web tool, consisting of a genomic prediction of secondary metabolomes. Using different algorithms that compare the new genetic information with 57 virtual enzymatic reactions (such as adenylation, acyltransferase, and acyl-adenylating), this approach provides the possibility of obtaining a correspondence between known natural drugs and possible new ones [20]. (iii) “Integrated Microbial Genomes Atlas of Biosynthetic gene Clusters” (IMG/ABC) [21], launched in 2015, is a large open web database of known predicted microbial BGCs able to associate BGCs with secondary metabolites (SMs) and analyze both BGCs and SMs. In this way, it offers the ability of finding similar function between BGCs present in database and BGCs to be identified [22].

Starting from these general considerations, in the present review we want to emphasize the significance of genome mining approach to identify new natural products, also underlining the possible advantages and disadvantages of this technique. Moreover, we debate the associated techniques that can be applied following to genome mining for validation of data. Finally, we review the literature describing all novel natural drugs found from bacteria, fungi and other living organisms by genome mining approach, focusing on the examples available in the literature of the last ten years.

## 2. The Significance Genome Mining in Drug Discovery

Approximately half of clinically approved drugs (including antibiotics) are represented by natural products and their derivatives. Recently, the development of new bioinformatics, genetics and analytic tools, has provided new strategies for the discovery of natural products of biotechnological interest known as “combinatorial biosynthesis approaches” [23,24]. These techniques, together with bioinformatic approaches, have shown that the ability of organisms (particularly microorganisms) to produce bioactive natural products has been underestimated [25]. These organisms have been deeper explored through the sequencing of their genome and the application of genome mining approaches [26]. Genome analysis has shed light the presence of numerous biosynthetic gene clusters that could be involved in the synthesis of other secondary metabolites defined cryptic or orphan for their unknown origin [25].

Genome mining aims at predicting the genes that encode for new natural compounds of biotechnological interest by using several bioinformatic approaches [21]. The importance of genome mining is based on the urgent need to discover new drug entities due to the increased incidence of severe diseases (such as cancer) and the reduced efficacy of existing drugs [27]. Furthermore, the biosynthetic gene clusters contain elements that can be used to increase the production of both natural and engineered products by promoting costs reduction and their commercial use [26].

### 2.1. Strengths and Weaknesses of Genome Mining

As in the case of all approaches, also genome mining has strengths and weaknesses, summarized in Table 1. One of the advantages of using genome mining is to foster the detection of a large amount of bioactive natural compounds [6]. In addition, genome mining approach is relatively cheap and easy to apply in laboratory, and it requires no particular skills and/or experience of the operators [28]. Combining genome mining with genetic engineering techniques will make it possible to achieve maximum diversity of natural products [29]. This bioinformatic approach allows to predict the chemical structure of bioactive natural products, but forecasts are often difficult to formulate [28,29].

As reported in Wohlleben et al. [6], a great disadvantage of genome mining is that only known biosynthetic gene clusters can be identified [29]. Moreover, with this approach, it is not possible to predict the biological activities of the natural products identified [26]. However, genome mining is still an evolving technique [29], in fact, scientists are trying to improve this bioinformatic tool in order to reduce the limits of this approach.

### 2.2. Synthetic Biology and Other Experimental Techniques Associated with Genome Mining

Synthetic biology progresses have been possible thanks to the very recent advent of DNA sequencing and synthesis in molecular biology field. The distinguishable element of synthetic biology respect to the other traditional molecular biology approaches is represented by its focus on the design and construction of components which are core for example of enzymes and metabolic pathways [30]. These genomic assessments joined to microbial diversity provide the fundamental natural libraries for further engineering.

In this review we have not focused our attention on synthetic biology because a great number of reviews on the most obvious and popular applications of synthetic biology methodologies have been published from 2008 to today [30,31,32,33,34,35,36,37,38,39,40,41,42,43,44,45].

Natural product production using engineered microorganisms represent the more important application of synthetic biology in the biotechnological field for natural products. The most important of commercialized examples are represented by two compounds produced by fermentation in genetically modified yeast: The semisynthetic malaria drug artemisinin and the first consumer-market synthetic biology product, “natural” vanillin [46,47]. These successful application of synthetic biology opened new perspective in the exploration of microbes as sources of high-value compounds on an industrial scale.

Genome mining is followed by the identification of cryptic pathways using several strategies, known as “combinatorial biosynthesis”, which that can be used in order to create novel genetic combinations of structural biosynthetic genes. These methods consist of gene activation/inactivation and mutasynthesis approaches. Gene inactivation involves the creation of a mutant organism, in which the biosynthetic gene cluster becomes inactive, thus eliminating the production of metabolites. The comparison between mutant organisms can be made by high-performance liquid chromatography/mass spectrometry (HPLC-MS), revealing the natural product absent in the mutant organism [26]. Therefore, gene inactivation needs as evidence of cluster involvement in compound biosynthesis [24]. Secondary metabolites come from precursors of primary metabolism, and their over-production is related to an enhanced protein synthesis [48]. However, in some cases, there are genes that produce specific precursors not provided by the primary metabolism. These precursors are usually used as starting units for example to the production of polyketides synthases (PKS) or non-ribosomal peptide synthetases (NRPS), which in turn produce natural compounds. Inactivation of genes involved in the biosynthesis of these precursors leads to non-productive mutants that can be used for the biosynthesis of new compounds by mutasynthesis or mutational biosynthesis [23,24,26]. If some genes are silent, it would be impossible to produce and test the biological activity of the natural product. It is therefore necessary to apply the activation of silent pathways under the control of a constitutive promoter or inactivating repressors [28]. In the final stages of metabolites biosynthesis, several enzymes such as, transferase, oxygenase, oxidase, peroxidase, and reductase, play a key role for further modifications [26].

#### Examples of Other Experimental Techniques

A method to identify new natural products with biotechnological potential combines the research of coding genes for a specific compound with the detection of bacterial resistance. This approach, called target-directed genome mining, relies on the identification of gene clusters without knowing the molecules produced [49].

Another method to identify a natural product is the one strain/many compounds (OSMAC) approach. This method is based on the systematic alteration of culture media or cultivation parameters to force the expression of cryptic genes. In addition, any metabolism deregulation system can be used to improve the production of secondary metabolites, leading to the discovery of new bioactive compounds. Many of these approaches involve the treatment of known chemicals that modify the structure of chromatin or the use of small molecules that re-shape and regulate secondary metabolism by inhibiting the synthesis of fatty acids [50].

Another technique associated with genome mining is the in vitro reconstruction of biosynthetic pathways that produce natural products. This technique can be used to generate highly pure intermediates, limiting side reactions such as the formation of toxic compounds and reducing protein-protein interactions [51].

Taking into account this background, we review on the new natural drugs found from bacteria, fungi and other living organisms by genome mining approach. We analyzed organisms that derive not only from marine environment but also from the terrestrial ones, considering that the genome mining and other techniques associated with it are still at the beginning for the discovery of bioactive compounds from the sea.

### 2.3. Bacteria

The first point that must be underlined is that the most of medicinal products described above derive from bacteria [6] (see Table 2). In fact, the available literature on genome mining mainly concerns these microorganisms. Specifically, soil and marine bacteria, such as actinomycetes, produce the greatest part of natural drugs identified in the last thirty years [52]. The actinomycetes can be isolated from various habitats, such as soil, sea deposits, sponges, corals, mollusks, seagrasses, and mangroves [53].

Hornung et al. [54] applied genome mining to identify strains capable of producing halogenase enzymes, where halogenations represent an important feature for the biological activity of a great number of different natural products. *Escherichia coli* DH5a and *E. coli* XL1 Blue were used to identify the complete halogenase gene sequence and to build primer-specific probes for these genes. Moreover, genomic DNA was isolated from 550 strains of actinomycetes available in strain collections. Using specific primer probes, it has been demonstrated that some actinomycetes are able to produce halogen enzymes.

Furthermore, nuclear magnetic resonance spectroscopy (NMR spectroscopy) was applied to understand the structure of these molecules, revealing that they were not exactly like those already known in literature. *Streptomyces*, a type of actinomycetes gram-positive bacteria, have also extensively been studied.

In fact, McAlpine et al. [55] used the genome mining approach to identify new antibiotic ECO-02301, with a potent antifungal activity, from *Streptomyces aizunensis* NRRL B-11277 bacteria. This compound was active against several strains of human pathogenic fungi (*Candida albicans* ATCC10231, *Candida glabrata* ATCC 90030, *Candida lusitaniae* ATCC 200953, *Candida tropicalis* ATCC 200955, *Candida krusei* LSPQ 0309, *Saccaromyces cerevisiae* ATCC 9763, *Aspergillus fumigatus* ATCC 204305, *Aspergillus flavus* ATCC 204304, *Cryptococcus neoformans* ATCC 32045). To obtain the expression of this gene after the grown of bacteria in flask, the proteins were extracted and analyzed by high performance liquid chromatography (HPLC) monitored by a diode array detector (DAD) that detects the absorption in UV region and positive/negative mass spectrometry (MS) ions.

The analysis of the genome of *S. aizunensis* NRRL B-11277 helped the prediction of the structure of this compound with sufficient accuracy so to represent a guide for its isolation.

Furthermore, an anti-infective agent, called arylomycin, and its BGCs by *Streptomyces roseosporus* strains, were described using imaging mass spectrometry (IMS) and MS guided by genome mining approaches [56]. Specifically, *S. roseosporus* was co-cultured with two pathogen strains, *Staphylococcus aureus* and *Staphylococcus epidermidis*, and its genome has been sequenced. It was so demonstrated that *S. roseosporus* produced daptomycin, an antibiotic molecule. Moreover, they spotted *S. roseosporus* in the center of *S. aureus* and *S. epidermidis* cultures and after 36 hours of incubation, using IMS and MS, aptomycin ions have been not observed, but a cluster corresponding to the potassium adduct was found. These results suggested that *S. roseosporus* was also able to produce three additional antibiotics. Furthermore, to identify the biosynthetic gene cluster of these molecules, a peptide-genomic mining approach was applied, which relied on the short sequence tag (SST) from tandem very spectrometric data. With this approach, in fact, they established that these three molecules were arylomycins.

In a similar study, Liu et al. [57] demonstrated that *S. roseosporus*, in addition to the non-ribosomal peptide synthetase-derived molecular families and their gene subnetworks, todaptomycin, arylomycin, and napsamycin, was also able to produce stenothricin. Firstly, after DNA extraction, to identify the molecular network they reduced the complexity of analysis to 837 genes using MS/MS spectra with parent ion masses within 0.3 Da and compared to related MS/MS spectral patterns. It was possible to observe the already known genes ofarylomycin, napsamycin, daptomycin and their variants. However, they identified four genes for stenothricin but combining the MS/MS spectra to the amino-acid blocks found by antiSMASH, 21 genes clusters were found. Furthermore, to understand their biological activities, a screening platform (named BioMAP) was used and then the cytological profiling, evaluating this activity against 15 bacterial strains. These approaches revealed that the stenothricinis was active on both Gram-negative and Gram-positive bacteria.

Seo et al. [58] used the DNA extraction to isolate the antibiotic pentalenolactone biosynthetic gene clusters from the known pentalenolactone producers *Streptomyces exfoliatus* UC5319 and *Streptomyces arenae* TU469. By building probes based on the previously cloned *S. exfoliates* pentalenene synthase gene, the sequence of the *S. exfoliatus* Pen biosynthetic gene cluster were analyzed, revealing that the furthest upstream gene, designated as PenR, encoded a 153 aa MarR-family transcriptional regulator. Moreover, PenI, PenH, and PenF were also found, which were expected to catalyze the oxidative conversion of pentalenene to 1-deoxy-11-oxopentalenic acid, as previously established for the othologous *Streptomyces avermitilis* proteins. Furthermore, the attention was pointed out on penE product, because it seems to be the key branch point that distinguished the pentalenolactone and neopentalenolactone biosynthetic pathways. PenE gene encoded a protein that is a homologue of the known Baeyer-Villiger monooxygenase from *S. avermitilis*, PtlE. The compounds PenD, PntD, and PtlD were characterized by mass spectrometry and H-NMR, also generating the deletion mutants with no production of pentalenolactone.

In another study, Tang et al. [49] analyzed, through bioinformatic approaches (BLASTP, Artemis Release 12.0), the genome of *Streptomycetes* sp. M10 discovering 20 biosynthetic gene clusters involved in the synthesis of natural products, such aspolyketides, NRPs, siderophores, lantibiotics, terpenoids. In addition, one of all gene clusters shared a partial similarity with candicidin/FR-008gene cluster, which in turn encoded for antifungal polyene assuming the potential role of this strain to produce this compound. Finally, to confirm this potential activity, the polyene was tested against the phytopathogen *Fulvia fulva* for its antifungal activity.

A high throughput genomic library expression analysis system (LEXAS) was applied for efficient, function-driven discovery of cryptic and new antibiotics from *Streptomyces*, known producers of several antibiotics [60]. Each BAC clone was transferred individually into an engineered antibiotic overproduction host, avoiding preference for smaller BACs. The LEXAS captured two known antibiotics, identified two novel lipopeptides and their BGC that was not produced/expressed in the native *Streptomyces rochei* strain, and revealed a cryptic BGC for unknown antibiotic. Specifically, in this research two new antibiotics streptothricins and borrelidin were found and for their validation these genes were expressed in the surrogate host *Streptomyces lividans* SBT5 by heterologous expression. Moreover, to analyze the antimicrobial activity, SBT5 products were tested against *Staphylococcus aureus* and *Bacillus mycoides*, showing an inhibition. In addition, they discovered two novel linear lipopeptides and their BGCs also adding the analysis of their structures by HPLC and liquid chromatography-mass spectrometry (LC-MS).

Thirty-eight secondary biosynthetic gene clusters of nataxazole (NAT) and its derivatives were identified from *Streptomyces* sp. Tü 6176, using in silico by genome mining and antiSMASH 2.0 [61]. In particular, the NAT entire BGC was described, consisting of 21 genes: 12 encoding for structural proteins, 4 for regulatory proteins, 4 probably involved in NAT secretion, and 1 with unknown function. Moreover, using the gene inactivation and heterologous expression of NAT cluster, it was established that secondary metabolite pathways were outside of NAT gene cluster (not a common in actinomycetes) despite they were involved in NAT biosynthesis. Furthermore, using antibiotic disc diffusion assay, an antibiotic activity was found only against *Staphylococcus albus* J1074, whereas the negative effect was absent in *Streptomyces lividans* JT46, *Micrococcus luteus* and *Escherichia coli*. Anticancer activity was tested against human tumor cell lines (HT29, A549, MDA-MB-231, AGS and A2780) including mouse cell line NIH/3T3 used as control. In this way, they demonstrated that these compounds have moderate activity against maleficent cells. In a similar study, Ye et al. (2017) used genome mining and antiSMASH 2.0 to identify the presence of 31 biosynthetic clusters in *Strepmomyces argillaceus* ATCC12956.

The most studied BGC between all found was the gene that encoded for argimycin P (renamed *arp* cluster). In addition, the pathway for the biosynthesis of *arp* was reconstructed by means of genetic engineering. Moreover, using in vitro tests on cells, no cytotoxic activity of this compound was found against 59 tumour cell lines. In another study, Paulo et al. [63] used in silico genome mining on strains of *Streptomyces* sp. CBMAI 2042, isolated from the branches of the plants *Citrus sinensis*. Moreover, this strain also prevented the proliferation of pathogens in citrus such as *Citrus xylella*, *Geotrichum candid* var. citri-Aurantii, and *Colletotrichum gloesporioides*. In particular, 35 biosynthetic gene clusters were found including the putative NRP biosynthetic gene cluster that encoded for valinomycin. In addition, by combining genome mining and molecular network, it was possible to reconstruct the origin of the biosynthetic pathway of cyclodepsipeptides, which have antibacterial, antiviral, and anticancer activity.

Furthermore, Purves et al. [64] applied the genome mining approach on bacteria extracted from two marine sediments (Antarctic and Scotland). They identified eight genera (*Bacillus*, *Streptomyces*, *Micromonospora*, *Paenibacillus*, *Kocuria*, *Verrucosispora*, *Staphylococcus*, and *Micrococcus*) and used 38 strains on which MS analysis was conducted. Thanks to this approach a great number of metabolites were identified, of which 1422 were Antarctic-specific, while 1501 were Scottish-specific secondary metabolites. Moreover, a molecular network was built up by Global Natural Products Social (GNPS) Molecular Networking, showing that only 8% of strains belonging to these locations displayed a similarity, implying a high degree of biogeographic influence upon secondary metabolite production. Organic extracts from these 38 selected strains were tested for cytotoxicity against epithelial colon adenocarcinoma cells (Caco-2) and human fibroblasts originating from foreskin (HS27). No effect on normal cell viability was observed, while seven extracts were bioactive against Caco-2 at 50 g/mL concentration. Direct observation revealed morphological changes, such as cell shrinkage and formation of apoptotic bodies. Moreover, Deng et al. [65] identified three new fluorinase enzymes from three bacterial strains, *Streptomyces* sp. MA37, *Nocardia brasiliensis*, and *Actinoplanes* sp. using the genome mining approach. These proteins were isolated and purified using overexpression of fluorinasegenes in *Escherichia coli*. Analyzing this product with in vitro activity assay, it revealed a high homology (about 85%) of its BGCs to the original one (called flA1) founded in *Streptomyces cattleya*. Finally, it was also assessed that *Streptomyces* sp. MA37produced some unidentified fluorometabolites.

As mentioned before, the actinomycetes are distributed in different marine habitats, being mainly associated to sponges. In fact, Jin et al. [53] have conducted genome mining experiments with *Streptomyces* sp. PKU-MA00045 isolated from sponges. Specifically, five new aromatic polyketides, fluostatins M–Q (1–5) were isolated using PCR-based genome mining method, and their chemical structures were clarified by ^1^H-NMR and ^13^C-NMR. The entire genome of *Streptomyces* sp. PKU-MA00045 was sequenced and compared to homologues in the published fluostatin gene clusters with BLAST, so identifying the BGCs of these new five compounds. In a similar experiment, Almeida et al. [10] used OSMAC approach to identify an octapeptidicsurugamide (Surugamide A) from *Streptomyces* sp. SM17, isolated from the marine sponge *Haliclona simulans.* The phylogenetic analysis with NCBI BLASTN demonstrated that this marine bacteria was phylogenetically linked to five strains of terrestrial *Streptomyces* bacteria: *Streptomyces albidoflavus* strain J1074, *Streptomyces albidoflavus* strain SM254, *Streptomyces sampsonii* strain KJ40, *Streptomyces* sp. FR-008 and *Streptomyces koyangensis* strain VK-A60T. Since *S. albidoflavus* strain J1074 was widely used as a model for various biotechnological studies, the secondary metabolites of the biosynthetic gene clusters were predicted by antiSMASH program, comparing the new BGCs with those already collected by *S. albidoflavus* strains. In this way, it was demonstrated that *Streptomyces* sp. SM17 produced different secondary metabolites. Moreover, using NMR technique it was possible to show that *Streptomyces* sp. SM17 was able to produce higher levels of Surugamide A than the *S. albidoflavus* strain J1074.

However, Anoop et al. [66] studied another bacterial strain *Pseudovibrio* sp. POLY-S9, isolated from intertidal marine sponge *Polymastia penicillus* sampled from the Atlantic coast of Portugal. In fact, after genome sequencing of this marine bacteria, new genes-related bioactive compounds were isolated, such as polyketide synthase, nonribosomal peptide synthetase and siderophore, using genome mining by antiSMASH. Moreover, several genes involved in symbiotic relationships, such as the ankyrin repeats, tetratrico peptide repeats and Sel1, were also identified. Another important finding of this study was represented by some genome plasticity elements of POLYS9, which allowed the survival of these bacteria and their adaptation to various habitats through the exchange of genetic material. Using MS/MS-based molecular networking analysis a bacterial strain was isolated from the Caribbean sponge *Tectitethya crypta*, able to produce spongosine, deoxyspongosine, spongothymidine, and spongouridine, generally referred as “spongonucleosides” [67].

Spongosine, a methoxyadenosine derivative, had several pharmacological applications, having anti-inflammatory activity (for their capability to inhibit the nitric oxide production in cells) and analgesic and vasodilation properties. After MLSA and BLAST analyses, this strain was identified as *Vibrio harveyi*, and thanks the genomic DNA sequencing and antiSMASH platform, six potential secondary metabolite pathways were described.

Planctomycetes are ubiquitous bacteria that were usually found in marine, freshwater and soil habitats, even if it is possible to find them as free living organisms, or attached to abiotic and biotic surfaces, as for example to algal cells. Some strains also live as symbionts of prawns, marine sponges or termites [72]. For instance, Jeske et al. [68] applied the genome mining methods to define the metabolic properties of *Planctomyces*. First, they found 102 genes or gene clusters involved in the production of secondary metabolites by analyzing 13 genomes on antiSMASH database. Moreover, the genome analysis showed a close correlation between the length of BGCs and the amino acid sequence of the predicted secondary metabolites. Moreover, since most BGCs were transcriptional silent, the Phenotype MicroArray technology was applied on compounds secreted by *Planctomyces limnophilus* (limnic strain) and *Rhodopirellula baltica* (marine strain), confirming that there was a strong relationship between *Planctomycetes* and algae or plants, which in turn secrete compounds that might serve as trigger to stimulate the secondary metabolite production in *Planctomycetes*. Thus, this study provides strong evidences for the use of these bacteria for drug development.

In a different study, Guérard-Hélaine et al. [69] identified new aldolase enzymes, belonging the aldolase/transaldolase family, from 313 different prokaryote species. Comparing the sequence of 1148 proteins extracted from these strains to already known aldolases and transaldolases, 700 genes were selected. The overexpression of these genes and the following LC-MS analysis allowed the selection of 19 proteins of interest. After cloning of the corresponding genes and using fast protein liquid chromatography (FPLC), 18 enzymes were purified, including two aldolases and sixteen transaldolase. Moreover, the activity of these 18 enzymes was evaluated by high-throughput screening (HTS), revealing that six of those annotated as transaldolase showed aldolase activity. Maansson et al. [8] extracted DNA from 13 closely related strains identified as *Pseudoalteromonas luteviolacea*, isolated from all over the Earth, and analysed their potential to produce secondary metabolites. Specifically, antiSMASH analysis demonstrated that only 10 biosynthetic pathways were preserved in all strains, including glycosylated lantipeptide (RiPP1) and two bacteriocins (RiPP2 and RiPP3). All strains have maintained essential pathways, such as that responsible for the production of siderophores, homoserine lactones and violacein. Furthermore, bacteria were grown in culture media to stimulate the synthesis of secondary metabolites and the chemical structures of these compounds were analyzed by LC-MS/MS. Particular attention was paid on violacein pathway, showing the presence of an insert in the *bmp1* gene in the thioesterase domain probably responsible of *Pseuoalteromonas* color. Moreover, the varieties *Pseudoalteromonas* S4047-1, S4054 and CPMOR-1 produced indolmicin antibiotic. However, the biosynthetic pathway coding for the antibiotic indolmicin has never been characterized.

#### Cyanobacteria

Cyanobacteria were also studied for their interesting bioactive secondary metabolites. For example, they produce mycosporine and mycosporine-like amino acids (MAAs), which are antioxidant molecules that eliminate toxic oxygen radicals protecting cells from saline, drying or thermal stress in some organisms and may act as an intracellular nitrogen reservoir. These compounds were also found in many other organisms such as yeasts, fungi, algae, corals and lichens [73]. Applying genome mining approach and BLAST analysis, Singh et al. [70] demonstrated that among four strains of cyanobacteria (*Anabaena variabilis* PCC 7937, *Anabaena* sp. PCC 7120, *Synechocystis* sp. PCC 6803 and *Synechococcus* sp. PCC 6301) exposed to 72 hours of UV radiation, only *Anabaena variabilis* PCC 7937 was able to produce MAAs. HPLC analysis of these four cyanobacteria revealed the presence of a unique combination of two genes, predicted *DHQ synthase* (YP\324358) and *O-methyltransferase*; (YP\324357) in *A. variabilis* PCC7937, which were missing in other non-MAAs-synthesizing cyanobacteria. Micallef et al. [71] identified the gene cluster responsible for hapalosine synthesis and hapalosine biosynthetic pathway from the genomes of three cyanobacteria (*Hapalosiphon welwitschii* UH strain IC-52-3, *Westiella intricata* UH strain HT-29-1 and *Fischerella* sp. CC 9431), by using genome mining combined with Geneious version 6.1.7 and antiSMASH. Single cyanobactin cluster of biosynthetic genes was identified only in the genome of *W. intricate* UH strain HT-29-1, demonstrating that there is structural diversity of cyanobacteria inside cyanobacteria strains. Moreover, only *Fischerella* sp. PCC 9339 encoded a microviridine gene cluster and they identified the MAA (*mys*) gene cluster in the strains *W. intricate* varieties UH HT-29-1, *H. welwitschii* UH strain IC-52-3, *Mastigocoleus testarum* BC008, *Fischerella muscicola* SAG1427–1 and *Chloroglopsis* sp. PCC 9212. Finally, the presence of the cluster of scytonemin genes within the genome of *Mastigocladopsis repens* PCC 10,914 was discovered, suggesting that this organism was able to bio-sintetizes cytonemin in order to protect the cells against UVA-radiation. The geosmin gene cluster was identified in *W. intricata* variety UH HT-29-1, *H. welwitschii* UH strain IC-52-3, *Fischerella* sp. PCC 9431, and *F. muscicola* SAG 1427–1.

### 2.4. Fungi

As described above, the most important sources of natural drugs are not only bacteria but also fungi [6]. In fact, many different natural products, such as penicillin, cephalosporin, ergotrate and the statins represent well-known fungal secondary metabolites for pharmacological applications [74]. For these organisms the genome mining also proved to be a useful method to find BGCs (Table 3). In a study of Bergmann et al. [75] a silent metabolic pathway was detected, which might code for the biosynthesis of polyketides or polypeptides in *Aspergillus nidulans.* In particular, considering that the cryptic gene cluster provided a putative activator gene called *apdR*, it was amplified and cloned into expression vector pAL4, which coded for inducible alcohol dehydrogenase promoter *alcAp* of *A. nidulans* and the pyr-4 gene of *Neurospora crassa* as a selectable marker.

Using Southern blot analysis, it was demonstrated that under inducing conditions the *apdA* gene encoded the PKS-NRPS hybrid synthetase. Moreover, HPLC analysis displayed that this induced strains were able to produce two main products, Aspyridones A and B, and two minor compounds, whose structures was elucidated by NMR and MS. In a similar study, Mao et al. [76] revealed a silent metabolic pathway involved in natural product biosynthesis. In fact, after genome sequencing, 68 BGCs were identified, being in contrast to the two predominant metabolites normally produced, the F1-ATPase inhibitors 1 and 2. Since these BGCs are localized within the heterochromatic regions, a mutant strain was built deleting *hdaA* (gene of the histone H3 lysine 14 (K14) deacetylase). In this way, using metabolite extraction and LC-MS analysis, it was demonstrated that the mutant produced more compounds compared to wild strain. Moreover, after overexpression of these genes, ten compounds were isolated, of which four contained new structures, including the cyclic peptides arbumycin and arbumelin, the diterpenoid arbuscullic acid A, and the meroterpenoid arbuscullic acid B. However, Ye et al. [78] applied the genome mining approach to conduct a phylogenetic analysis of fifteen bifunctional terpene synthases found in five fungal genomes. Specifically, the terpene BGCs sequence were different and synthetized sesterterpenes with new carbon skeletons, suggesting that these microorganisms were separated in five different clades. Moreover, two clades, *Aspergillus oryzae* and *Neosartorya fischeri*, did not produce terpene, hypothesizing that BGCs were silent in standard conditions. For these reasons, heterologous expression was performed in *A. oryzae* using *E. coli* plasmids and the extract was analyzed with GC-MS, ^1^H- and ^13^C-NMR elucidating the structure of four compounds, one of which known as sesterfisherolsynthase (*NfSS*) and previously found in *N. fischeri*. Furthermore, bioinformatic analysis showed that *NfSS* gene was encoded downstream of a cytochrome P450 monooxygenase (NfP450) and it was transformed by NfP450 to sesterfisheric acid. Finally, to identify NfP450 gene, double transformant with NfSS and NfP450 genes was prepared and the extract was examined by LC-MS and HR-MS indicating that NfP450 conducted a NfSS modification.

Furthermore, Ding et al. [77] have identified the first BGCs of the stephacidin and notoamide, belong to family of prenylated alkaloids, from *Aspergillus* sp. MF297-2. Specifically, after sequencing of genome the entirenotoamide and stephacidin gene cluster was identified by BLAST comparing sequence to gene *ftmA*, which was previously mined from an *Aspergillus fumigatus*. By bioinformatics analysis, 19 genes involved in notoamide biosynthesis were found to constitute this cluster. To understand the function, this cluster was cloned using *E. coli* DH5R and overexpressed into *E. coli* BL21. The proteins were purified with a single Ni-NTA column and analyzed with HPLC, LC-MS, ^1^H- and ^13^C-NMR. Two central pathway enzymes, *NotF* and *NotC,* were identified suggesting a scheme for the biosynthesis of stephacidin and notoamide metabolites.

### 2.5. Other Organisms

Several other organisms, completely unrelated to the marine environment, have been used as subject of genome mining approach, such as terrestrial microorganisms, plants, and animals (Liu et al. 2018; Table 4).

Gruber and Muttenthaler [4] applied genome mining to identify defense- and neuropeptides in the genomes of social ants of the subfamilies of Myrmicinae (*Atta cephalotes*), Formicinae (*Camponotus floridanus*) and Ponerina (*Harpegnathos saltator*); ants are difficult to manipulate for scientific purposes because of the size of their bodies and organs. Most interestingly, genes encoding for oxytocin/vasopressin-related peptides (inotocins) and their putative receptors were identified, using a publicly available matrix of tools, including the search for similarity with tBLASTn, prediction of gene structure using GeneWise algorithm and alignments of sequences by ClustalW.

Carotenoids cannot be synthesized de novo, but they must therefore be taken with food (such as algae) and get protective human health benefits as well. Free astaxanthin and its esterified forms are the main carotenoids present in crustaceans and in particular in copepods. Mojib et al. [79] aimed on understanding the metabolic and genetic basis of the blue phenotype between the blue pigmented organisms from the phylum Arthropoda, subclass Copepoda (*Acartia fossae*) and the phylum Chordata, class Appendicularia (*Oikopleura dioica*) in the Red Sea. Firstly, liquid chromatography-UV method was used to detect the carotenoids and mass spectrometry and HPLC were used to detect intermediate metabolites, present at low concentrations. The chromatograms identified astaxanthin in all samples, while the fucoxanthin was not detected in any samples. In addition, other carotenoids, intermediate compounds for conversion from β-carotene to astaxanthin, were also identified. The metabolic pathway for each sample was reconstructed for the conversion from β-carotene to astaxanthin. The results showed that all the species followed the same metabolic pathways via almost the same intermediate metabolite formation. Echinenone, one of the intermediate metabolite was not detected in any of the samples but its hydroxylated form, the 3-idrossi chinenone, was detected in all samples, as well as lutein. Putative β-carotene hydroxylase of P450 family coding transcripts was identified in blue *A. fossae* by in silico transcriptome mining. Putative carotenoid-binding proteins after transcriptome/genome mining showing 100% homology to Apolipoprotein D (ApoD) and crustacyanin as predicted by HHpred database.

A customized version of the plantiSMASH genome mining algorithm was created to identify a sesterterpene synthase gene repertoire in some *Brassicaceae* plants, which synthesizes fungal-type sesterterpenes with diverse scaffolds, thus fueling the drug-discovery pipeline [80]. Sesterterpenoids are a rare terpene class with not well explored chemical structure and diversity, representing a potential new drug source. This study offered new insights on the origin of structural diversity for protein engineering, supporting the idea of convergent evolution for natural product biosynthesis.

## 3. General Conclusions

Many drugs used, for example, as anticancer, antibacterial, and anti-inflammatory agents in the clinical field are derived from natural products and their derivatives. In fact, these secondary metabolites are produced by all organisms (from bacteria to plants, invertebrates, and other animals) and show several biological activities useful in several biotechnological applications. However, the most important sources of natural drugs are microorganisms (mainly bacteria, also associated with marine organisms, such to sponges) and fungi. In the last decades, the great advances made in the field of molecular biology techniques, representing a good example the genome mining together with the synthetic biology, strongly push the identification of BGCs, encoding for enzymes involved in the biosynthesis of natural products. Taking together, these next-generation and highly sophisticated tools contribute to the emergence of a new generation of natural product research. These techniques are in their infancy for their application to marine environment, but there are in literature a lot of applications for the discovery of bioactive natural products for other environments. For this reason, we think that a review reporting all these examples could give strong support in pushing the applications of these new techniques in discovery bioactive compounds from the marine environment, also due to high level of biodiversity offered by the sea in comparison with the Earth. Genome mining, as well as synthetic biology and all the techniques to them associated, represent a new challenge in natural products discovery from the marine environment, without impact on the environment and with no use of destructive collection practices of marine organisms.

## Figures and Tables

**Table 1:** Strengths and weakness in the use of genome mining.

Strengths	Weaknesses
Easy to apply for experimental procedures in laboratory	Not to predict biotechnological potential of the natural compounds
Cheap and easy to apply in laboratory	Only known biosynthetic gene clusters
To predict chemical structures of bioactive natural products	Difficulty to formulate chemical structures
No particular skills and/or experience of the operators	Too new approach that needs to be deepened

**Table 2:** Genome mining approaches applied to microorganisms.

Microorganism	Experimental Purpose	Associated Techniques	References
*Actinomycetes*	Identification of strains capable to produce halogen enzymes.	PCR screening and NMR spectroscopy	[54]
*Streptomyces aizunensis* NRRL B-11277	Elucidation of new antibiotic ECO-02301 structure	HPLC, MIC	[55]
*Streptomyces roseosporus*	Anti-infective agent arylomycin and its BGCs	IMS, MS and SST	[56]
*Streptomyces roseosporus*	Identification of stenothricin and its BGCs	MS/MS spectra, antiSMASH, NMR, BioMAP, Cytological profiling	[57]
*Streptomyces exfoliatus* UC5319, *Streptomyces arenae* TU469 and *Streptomyces avermitilis*	Biosynthetic gene clusters involved in the synthesis of pentalenolactone	Cloning, MS/MS spectra, H-NMR spectroscopy	[58]
*Streptomycetes* sp. M10	To determine biosynthetic gene clusters involved in the synthesis of natural products	PRC screening, BLASTP, antiSMASH, Artemis Release 12.0, RT-PCR, MALDI-TOF	[59]
*Streptomyces* sp., *Streptomyces roche*, *Streptomyces lividans* SBT5	Streptothricin and borrelidin biosynthetic gene clusters	Heterologous expression, HPLC, LC-MS, LEXAS method, antiSMASH	[60]
*Streptomyces* sp. Tü 6176:	BGCs of nataxazole	antiSMASH 2.0 heterologous expression, gene inactivation, antibiotic disc diffusion assay, test on cancer cell lines	[61]
*Strepmomyces argillaceus* ATCC12956	Argimycin biosynthetic gene cluster	AntiSMASH, test on cancer cell lines	[62]
*Streptomyces* sp. CBMAI 2042	Valinomycin biosynthetic gene cluster	Test on pathogens, *in silico* analyses	[63]
*Bacillus*, *Streptomyces*, *Micronospora*, *Paenibacillus*, *Kocuria*, *Verricosispora*, *Staphylococcus*, *Micrococcus*	Influence of isolation location on secondary metabolite production	Test on cancer cell lines, MS, GNPS	[64]
*Streptomyces* sp. MA37, *Norcardia brasiliensis*, *Actinoplanes* sp. N902-109	Identification of Fluorinases	overexpression of gene, vitro activity assay and 19F NMR	[65]
*Streptomyces* sp. PKU-MA00045	Aromatic polyketides	1H-NMR and 13C-NMR spectra, genome sequencing, BLAST	[53]
*Streptomyces* sp. SM17	Identification of Surugamide A	NCBI BLASTN, antiSMASH, NMR	[10]
*Pseudovibrio* sp. POLY-S9	BGCs of symbiotic bacteria and gene involved in symbiontic relationship	genome sequencing, antiSMASH	[66]
*Vibrio harveyi*	BGCs of spongosine and potential secondary metabolites	MS/MS-based molecular networking, nitric oxide assay, MLSA and BLAST, genome sequencing and antiSMASH	[67]
*Planctomyces*	Metabolic properties of these bacteria	antiSMASH, MicroArray	[68]
*Diverse prokaryotic species*	New aldolase enzymes	LC–MS, cloning, FPLC, HTS	[69]
*Pseudoalteromonas luteviolacea*	Violacein biosynthetic pathway	LC-MS/MS, antiSMASH	[8]
*Anabaena variabilis* PCC 7937, *Anabaena* sp. PCC 7120, *Synechocystis* sp. PCC 6803 and *Synechococcus* sp. PCC 6301	MAA biosynthetic gene cluster	MAA induction with radiation UVR, MAA extraction, HPLC, BLAST	[70]
*Hapalosiphon welwitschii UH* strain IC-52-3, *Westiella intricate UH* strain HT-29-1 and *Fischerella* sp. CC 9431	Hapalosine biosynthetic pathway	PCR screening, antiSMASH, Geneious version 6.1.7	[71]

**Table 3:** Genome mining approaches applied to fungi.

Fungi	Experimental Purpose	Associated Techniques	References
*Aspergillus nidulans*	Detection of silent metabolic pathway	Southern blot, HPLC, NMR, IR, and MS	[75]
*Calcarisporium arbuscula*	Silent metabolic pathway involved in natural product biosynthesis	genome sequencing, LC-MS, chromatographic and NMR analysis, HPLC	[76]
*Aspergillus* MF297-2	Identification of BGCs of ephacidin and notoamide	genome sequencing, BLAST, gene cloning, overexpression of protein, HPLC, LC-MS, 1H, and 13C NMR	[77]
*Aspergillus oryzae* and *Neosartorya fischeri*	Isolation of terpene synthases	heterologous expression, GC-MS, 1H- and 13C-NMR, LC-MS, and HR-MS	[78]

**Table 4:** Genome mining approaches applied to ants, copepods and plants.

Organism	Experimental Purpose	Associated Techniques	References
*Atta cephalotes*, *Camponotus floridanus* and *Harpegnathos saltator*	Defense- and neuropeptides in Social Ants	tBLASTn, GeneWise algorithm, ClustalW	[4]
*Calanus* sp., *Pontella* sp., *Oikopleura* sp., *Acartia* sp., *Acartia* sp. and *Corycaeus* sp.	Metabolic pathway from conversion from β-carotene to astaxanthin.	LC-UV method, HPLC, Hhpred database	[79]
*Arabidopsis thaliana*, *Capsella rubella*, *Brassica oleracea*, *Nicotiana benthamiana*, *Agrobacterium tumefaciens*	Sesterterpene biosynthetic gene cluster	plantiSMASH, heteroloug expression, GC-MS, cristallography	[80]

## References

[1] Ren H., Wang B., Zhao H. (2017). Breaking the silence: New strategies for discovering novel natural products. Curr. Opin. Biotechnol..

[2] Newman D.J., Cragg G.M. (2014). Marine-sourced anti-cancer and cancer pain control agents in clinical and late preclinical development. Mar. Drugs.

[3] Gruber C.W. (2010). Global cyclotide adventure: A journey dedicated to the discovery of circular peptides from flowering plants. Pept. Sci..

[4] Gruber C.W., Muttenthaler M. (2012). Discovery of defense- and neuropeptides in social ants by genome-mining. PLoS ONE.

[5] Boddy C.N. (2014). Bioinformatics tools for genome mining of polyketide and non-ribosomal peptides. J. Ind. Microbiol. Biotechnol..

[6] Wohlleben W., Mast Y., Stegmann E., Ziemert N. (2016). Antibiotic drug discovery. Microb. Biotechnol..

[7] Peláez F. (2006). The historical delivery of antibiotics from microbial natural products—Can history repeat?. Biochem. Pharmacol..

[8] Maansson M., Vynne N.G., Klitgaard A., Nybo J.L., Melchiorsen J., Nguyen D.D., Sanchez L.M., Ziemert N., Dorrestein P.C., Andersen M.R. (2016). An integrated metabolomic and genomic mining workflow to uncover the biosynthetic potential of bacteria. Am. Soc. Microbiol..

[9] Gulder T.A.M., Moore B.S. (2009). Chasing the treasures of the sea-bacterial marine natural products. Curr. Opin. Microbiol..

[10] Almeida E.L., Kaur N., Jennings L.K., Felipe A., Rinc C., Jackson S.A., Thomas O.P., Dobson A.D.W. (2019). Genome mining coupled with OSMAC-based cultivation reveal differential production of Surugamide A by the marine sponge isolate *Streptomyces* sp. SM17 when compared to its terrestrial relative *S. albidoflavus* J1074. Microorganisms.

[11] Tracanna V., De Jong A., Medema M.H., Kuipers O.P. (2017). Mining prokaryotes for antimicrobial compounds: From diversity to function. FEMS Microbiol. Rev..

[12] Durand G.A., Raoult D., Dubourg G. (2019). Antibiotic discovery: History, methods and perspectives. Int. J. Antimicrob. Agents.

[13] Nett M. (2014). Genome mining: Concept and strategies for natural product discovery. Prog. Chem. Org. Nat. Prod..

[14] Challis G.L. (2008). Genome mining for novel natural product discovery. J. Med. Chem..

[15] Ziemert N., Alanjary M., Weber T. (2016). The evolution of genome mining in microbes—A review. Nat. Prod. Rep..

[16] Trivella D.B.B., De Felicio R. (2018). The tripod forbacterial natural product discovery: Genome mining, silent pathway induction, and mass spectrometry-based molecular networking. mSystems.

[17] Koonin E.V. (2009). Evolution of genome architecture. Int. J. Biochem. Cell Biol..

[18] Timmermans M.L., Paudel Y.P., Ross A.C. (2017). Investigating the biosynthesis of natural products from marine proteobacteria: A survey of molecules and strategies. Mar. Drugs.

[19] Blin K., Medema M.H., Kazempour D., Fischbach M.A., Breitling R., Takano E., Weber T. (2013). antiSMASH 2.0—A versatile platform for genome mining of secondary metabolite producers. Nucleic Acids Res..

[20] Skinnider M.A., Dejong C.A., Rees P.N., Johnston C.W., Li H., Webster A.L.H., Wyatt M.A., Magarvey N.A. (2015). Genomes to natural products PRediction Informatics for Secondary Metabolomes (PRISM). Nucleic Acids Res..

[21] Machado H., Tuttle R.N., Jensen P.R. (2017). Omics-based natural product discovery and the lexicon of genome mining. Curr. Opin. Microbiol..

[22] Hadjithomas M., Chen I.A., Chu K., Huang J., Ratner A., Palaniappan K., Andersen E., Markowitz V., Kyrpides N.C., Ivanova N. (2017). IMG-ABC: New features for bacterial secondary metabolism analysis and targeted biosynthetic gene cluster discovery in thousands of microbial genomes. Nucleic Acids Res..

[23] Helfrich E.J.N., Reite S., Piel J. (2014). Recent advances in genome-based polyketide discovery. Curr. Opin. Biotechnol..

[24] Olano C., Méndez C., Salas J.A. (2011). Molecular insights on the biosynthesis of antitumour compounds by actinomycetes. Microb. Biotechnol..

[25] Nett M., Ikeda H., Moore B.S. (2009). Genomic basis for natural product biosynthetic diversity in the actinomycetes. Nat. Prod. Rep..

[26] Olano C., Méndez C., Salas J.A., Villa T.G., Veiga-Crespo P. (2014). Strategies for the design and discovery of novel antibiotics using genetic engineering and genome mining. Antimicrobial Compounds: Current Strategies and New Alternatives.

[27] Mody K.H., Haldar S., Kim S.K. (2015). Genome mining for bioactive compounds. Springer Handbook of Marine Biotechnology.

[28] Scheffler R.J., Colmer S., Tynan H., Demain A.L., Gullo V.P. (2013). Antimicrobials, drug discovery, and genome mining. Appl. Microbiol. Biotechnol..

[29] Zerikly M., Challis G.L. (2009). Strategies for the discovery of new natural products by genome mining. ChemBioChem.

[30] Breitling R., Takano E. (2015). Synthetic biology advances for pharmaceutical production. Curr. Opin. Biotechnol..

[31] Keasling J.D. (2008). Synthetic biology for dynthetic chemistry. ACS Chem. Biol..

[32] Quin M.B., Schmidt-Dannert C. (2014). Designer microbes for biosynthesis. Curr. Opin. Biotechnol..

[33] Singh V. (2014). Recent advancements in synthetic biology: Current status and challenges. Gene.

[34] Sleator R.D. (2014). The synthetic biology future. Bioengineered.

[35] Unkles S.E., Valiante V., Mattern D.J., Brakhage A.A. (2014). Synthetic biology tools for bioprospecting of natural products in eukaryotes. Chem. Biol..

[36] Wilson M.C., Piel J. (2013). Metagenomic approaches for exploiting uncultivated bacteria as a resource for novel biosynthetic enzymology. Chem. Biol..

[37] Wright G. (2014). Synthetic biology revives antibiotics. Nature.

[38] Medema M.H., Breitling R., Bovenberg R., Takano E. (2010). production in microorganisms. Nat. Publ. Gr..

[39] Cobb R.E., Luo Y., Freestone T., Zhao H., Zhao H. (2013). Drug discovery and development via synthetic biology. Synthetic Biology—Tools and Applications.

[40] Hranueli D., Starcevic A., Zucko J., Rojas J.D., Diminic J., Baranasic D., Gacesa R., Padilla G., Long P.F., Cullum J. (2013). Synthetic biology: A novel approach for the construction of industrial microorganisms. Food Technol. Biotechnol..

[41] Zakeri B., Lu T.K. (2012). Synthetic biology of antimicrobial discovery. ACS Synth. Biol..

[42] Cummings M., Breitling R., Takano E. (2014). Steps towards the synthetic biology of polyketide biosynthesis. FEMS Microbiol. Lett..

[43] Genilloud O. (2014). The re-emerging role of microbial natural products in antibiotic discovery. Antonie Van Leeuwenhoek.

[44] Luo Y., Cobb R.E., Zhao H. (2014). Recent advances in natural product discovery. Curr. Opin. Biotechnol..

[45] Porro D., Branduardi P., Sauer M., Mattanovich D. (2014). Old obstacles and new horizons for microbial chemical production. Curr. Opin. Biotechnol..

[46] Paddon C.J., Keasling J.D. (2014). Semi-synthetic artemisinin: A model for the use of synthetic biology in pharmaceutical development. Nat. Rev. Microbiol..

[47] Kurita K.L., Glassey E., Linington R.G. (2015). Integration of high-content screening and untargeted metabolomics for comprehensive functional annotation of natural product libraries. Proc. Natl. Acad. Sci. USA.

[48] Wang G., Hosaka T., Ochi K., Wang G., Hosaka T., Ochi K. (2008). Dramatic activation of antibiotic production in *Streptomyces coelicolor* by cumulative drug resistance mutations. Appl. Environ. Microbiol..

[49] Tang X., Li J., Milla N., Zhang J.J., Neill E.C.O., Ugalde J.A., Jensen P.R., Mantovani S.M., Moore B.S. (2015). Identification of thiotetronic acid antibiotic biosynthetic pathways by target-directed genome mining. ACS Chem. Biol..

[50] Craney A., Ozimok C., Pimentel-elardo S.M., Capretta A., Nodwell J.R. (2012). Chemical perturbation of secondary metabolism demonstrates important links to primary metabolism. Chem. Biol..

[51] Kwon T., Lee G., Rhee Y., Park H., Chang M., Lee S., Lee J., Lee T. (2012). Identification of nickel response genes in abnormal early developments of sea urchin by differential display polymerase chain reaction. Ecotoxicol. Environ. Saf..

[52] Newman D.J., Cragg G.M. (2016). Natural products as sources of new drugs from 1981 to 2014. J. Nat. Prod..

[53] Jin J., Yang X., Liu T., Xiao H., Wang G., Zhou M., Liu F., Zhang Y., Liu D., Chen M. (2018). Fluostatins M–Q featuring a 6-5-6-6 ring skeleton and high oxidized A-rings from marine *Streptomyces* sp. PKU-MA00045. Mar. Drugs.

[54] Hornung A., Bertazzo M., Dziarnowski A., Schneider K., Welzel K., Wohlert S., Holzenkämpfer M., Nicholson G.J., Bechthold A., Süssmuth R.D. (2007). A genomic screening approach to the structure-guided identification of drug candidates from natural sources. ChemBioChem.

[55] Mcalpine J.B., Bachmann B.O., Piraee M., Tremblay S., Alarco A., Zazopoulos E., Farnet C.M. (2005). Microbial genomics as a guide to drug discovery and structural elucidation: ECO-02301, a novel antifungal agent, as an example. J. Nat. Prod..

[56] Liu W., Kersten R.D., Yang Y., Moore B.S., Dorrestein P.C. (2011). Imaging mass spectrometry and genome mining via short sequence tagging identified the anti-infective agent arylomycin in Streptomyces roseosporus. J. Am. Chem. Soc..

[57] Liu W., Lamsa A., Wong W.R., Boudreau P.D., Kersten R., Peng Y., Moree W.J., Duggan B.M., Moore B.S., Gerwick W.H. (2014). MS/MS-based networking and peptidogenomics guided genome mining revealed the stenothricin gene cluster in *Streptomyces roseosporus*. J. Antibiot. Tokyo.

[58] Seo M., Zhu D., Endo S., Ikeda H., Cane D.E. (2011). Genome mining in *Streptomyces*. Elucidation of the role of baeyer-villiger monooxygenases and non-heme iron-dependent dehydrogenase/oxygenases in the final steps of the biosynthesis of pentalenolactone and neopentalenolactone. Biochemistry.

[59] Tang J., Liu X., Peng J., Tang Y., Zhang Y. (2015). Genome sequence and genome mining of a marine-derived antifungal bacterium *Streptomyces* sp. M10. Appl. Microbiol. Biotechnol..

[60] Xu M., Wang Y., Zhao Z., Gao G., Huang S.-X., Kang Q., He X., Lin S., Pang X., Deng Z. (2016). Functional genome mining for metabolites encoded by large gene clusters using heterologous expression of a whole genomic BAC library in *Streptomyces*. Appl. Environ. Microbiol..

[61] Cano-prieto C., García-Salcedo R., Sánchez-Hidalgo M., Braña A.F., Fiedler H.-P., Méndez C., Salas J.A., Olano C. (2015). Genome mining of *Streptomyces* sp. Tü 6176: Characterization of nataxazole biosynthesis pathway. ChemBioChem.

[62] Ye S., Molloy B., Braña A.F., Zabala D., Olano C., Cortés J., Morís F., Salas J.A., Méndez C. (2017). Identification by genome mining of a Type I Polyketide gene cluster from *Streptomyces argillaceus* involved in the biosynthesis of pyridine and piperidine alkaloids Argimycins P. Front. Microbiol..

[63] Paulo B.S., Sigrist R., Angolini C.F.F., Oliveira L.G. (2019). De New cyclodepsipeptide derivatives revealed by genome mining and molecular networking. Chem. Sel..

[64] Purves K., Macintyre L., Brennan D., Hreggviðsson G.Ó., Kuttner E., Ásgeirsdóttir M.E., Young L.C., Green D.H., Edrada-ebel R., Duncan K.R. (2016). Using molecular networking for microbial secondary metabolite bioprospecting. Metabolites.

[65] Deng H., Ma L., Bandaranayaka N., Qin Z., Mann G., Kyeremeh K., Yu Y., Shepherd T., Naismith J.H., O’Hagan D. (2014). Identification of fluorinases from *Streptomyces* sp. MA37, *Norcardia brasiliensis*, and *Actinoplanes* sp. N902-109 by genome mining. ChemBioChem.

[66] Anoop A., Antunes A. (2015). Whole genome sequencing of the symbiont *Pseudovibrio* sp. from the intertidal marine sponge *Polymastia penicillus* revealed a gene repertoire for host-switching permissive lifestyle. Genome Biol. Evol..

[67] Bertin M.J., Schwartz S.L., Lee J., Korobeynikov A., Dorrestein P.C., Gerwick L., Gerwick W.H. (2014). Spongosine production by a *Vibrio harveyi* strain associated with the sponge *Tectitethya crypta*. J. Nat. Prod..

[68] Jeske O., Jogler M., Petersen J., Sikorski J., Jogler C. (2013). From genome mining to phenotypic microarrays: *Planctomycetes* as source for novel bioactive molecules. Antonie Van Leeuwenhoek.

[69] Guérard-Hélaine C., de Berardinis V., Besnard-Gonnet M., Darii E., Debacker M., Debard A., Fernandes C., Hélaine V., Mariage A., Pellouin V. (2015). Genome mining for innovative biocatalysts: New dihydroxyacetone aldolases for the Chemist’ s Toolbox. ChemCatChem.

[70] Singh S.P., Klisch M., Sinha R.P., Häder D. (2010). Genomics genome mining of mycosporine-like amino acid (MAA) synthesizing and non-synthesizing cyanobacteria: A bioinformatics study. Genomics.

[71] Micallef M.L., Agostino P.M.D., Sharma D., Viswanathan R., Moffitt M.C. (2015). Genome mining for natural product biosynthetic gene clusters in the Subsection V cyanobacteria. BMC Genom..

[72] Fuerst J.A., Sagulenko E. (2011). Beyond the bacterium: Planctomycetes structure and function. Nat. Rev. Microbiol..

[73] Oren A., Gunde-cimerman N. (2007). Mycosporines and mycosporine-like amino acids: UV protectants or multipurpose secondary metabolites?. FEMS Microbiol. Lett..

[74] Keller N.P., Turner G., Bennett J.W. (2015). Fungal secondary metabolism—from biochemistry to genomics. Nat. Rev. Microbiol..

[75] Bergmann S., Schümann J., Scherlach K., Lange C., Brakhage A.A., Hertweck C. (2007). Genomics-driven discovery of PKS-NRPS hybrid metabolites from *Aspergillus nidulans*. Nat. Chem. Biol..

[76] Mao X., Xu W., Li D., Yin W., Chooi Y., Li Y., Tang Y., Hu Y. (2015). Epigenetic genome mining of an endophytic fungus leads to the pleiotropic biosynthesis of natural products. Angew. Commun..

[77] Ding Y., De Wet J.R., Cavalcoli J., Li S., Greshock T.J., Miller K.A., Finefield J.M., Sunderhaus J.D., Mcafoos T.J., Tsukamoto S. (2010). Genome-based characterization of two prenylation steps in the assembly of the Stephacidin and Notoamide anticancer agents in a marine-derived *Aspergillus* sp.. J. Am. Chem. Soc..

[78] Ye Y., Minami A., Mándi A., Liu C., Taniguchi T., Kuzuyama T., Monde K., Gomi K., Oikawa H. (2015). Genome mining for sesterterpenes using bifunctional terpene synthases reveals a unified intermediate of di/sesterterpenes. J. Am. Chem. Soc..

[79] Mojib N., Amad M., Thimma M., Aldanondo N., Kumaran M., Irigoien X. (2014). Carotenoid metabolic profiling and transcriptome-genome mining reveal functional equivalence among blue-pigmented copepods and appendicularia. Mol. Ecol..

[80] Huang A.C., Kautsar S.A., Hong Y.J., Medema M.H., Bond A.D., Tantillo D.J., Osbourn A. (2017). Unearthing a sesterterpene biosynthetic repertoire in the Brassicaceae through genome mining reveals convergent evolution. Proc. Natl. Acad. Sci. USA.

